# Cardiopulmonary resuscitation (CPR) training strategies in the times of COVID-19: a systematic literature review comparing different training methodologies

**DOI:** 10.1186/s13049-021-00869-3

**Published:** 2021-03-29

**Authors:** Daniyal Mansoor Ali, Butool Hisam, Natasha Shaukat, Noor Baig, Marcus Eng Hock Ong, Jonathan L. Epstein, Eric Goralnick, Paul D. Kivela, Bryan McNally, Junaid Razzak

**Affiliations:** 1 Centre of Excellence Trauma and Emergencies, Aga Khan University, Karachi, Pakistan; 2 Department of Community Health Sciences, Aga Khan University, Karachi, Pakistan; 3 Department of Emergency Medicine, Aga Khan University, Karachi, Pakistan; 4 Department of Emergency Medicine, Singapore General Hospital, Singapore, Singapore; 5 Health Services and Systems Research, Duke-NUS Medical School, Singapore, Singapore; 6 Emergency Care Safety Institute, Public Safety Group, Burlington, MA USA; 7 Department of Emergency Medicine, Harvard Medical School, Boston, MA USA; 8 Department of Emergency Medicine, University of Alabama, Birmingham, USA; 9 Department of Emergency Medicine, Emory University, Atlanta, GA USA; 10 Centre of Global Emergency Care, Johns Hopkins University, Baltimore, USA

**Keywords:** CPR training methodologies, Basic life support (BLS), Standard CPR training, Alternative CPR training, Hybrid CPR training, Online CPR training, Layperson

## Abstract

**Background:**

Traditional, instructor led, in-person training of CPR skills has become more challenging due to COVID-19 pandemic. We compared the learning outcomes of standard in-person CPR training (ST) with alternative methods of training such as hybrid or online-only training (AT) on CPR performance, quality, and knowledge among laypersons with no previous CPR training.

**Methods:**

We searched PubMed and Google Scholar for relevant articles from January 1995 to May 2020. Covidence was used to review articles by two independent researchers. Effective Public Health Practice Project (EPHPP) Quality Assessment Tool was used to assess quality of the manuscripts.

**Results:**

Of the 978 articles screened, twenty met the final inclusion criteria. All included studies had an experimental design and moderate to strong global quality rating. The trainees in ST group performed better on calling 911, time to initiate chest compressions, hand placement and chest compression depth. Trainees in AT group performed better in assessing scene safety, calling for help, response time including initiating first rescue breathing, adequate ventilation volume, compression rates, shorter hands-off time, confidence, willingness to perform CPR, ability to follow CPR algorithm, and equivalent or better knowledge retention than standard teaching methodology.

**Conclusion:**

AT methods of CPR training provide an effective alternative to the standard in-person CPR for large scale public training.

## Background

Sudden Cardiac Death (SCD) refers to an unexpected death from cardiac arrest [1]. Worldwide, SCD is the most common cause of death accounting for 17 million deaths every year or 25% of all global mortality [1]. Out-of-hospital cardiac arrest (OHCA) is a global health issue with incidence reported as 40.6 per 100,000 person-years in Europe, 47.3 in North America, 45.9 in Asia, and 51.1 in Australia [2–5].

Decreasing the time to initiation of CPR is crucial for improving outcomes in cases of cardiac arrest [6, 7]. This is where the role of the bystander – any layrescuer (non-medical professional) who witnesses a medical emergency – comes into play [8]. In fact, bystander CPR before arrival of EMS is independently associated with up to a threefold increase in survival [9]. Various attempts have been made to increase the number of people trained in CPR and therefore improve bystander CPR rates, including organization of mass CPR training events. These attempts, particularly when backed by effective legislation mandating CPR training, result in significantly more laypersons trained in CPR as demonstrated by efforts led in Norway [10, 11], Singapore [12], and Denmark [13].

CPR has traditionally been taught face to face using a mannikin as a proxy for a patient. In 2015, the American Heart Association introduced the concept of blended learning that involved the use of online videos and simulated Voice Assisted Mannikins to replace instructors. CPR self-instruction through video- and/or computer-based modules paired with hands-on practice may be an effective alternative to instructor-led courses and such technologies can be utilized more easily to facilitate safe and effective learning [14, 15]. This has become particularly relevant now that the COVID-19 pandemic, where wide spread restrictions on in-class training and potential risk of virus spread during face-to-face sessions, has caused organizations to reconsider how trainings are allowed to be conducted [16, 17].

The aim of this systematic review is to compare the learning outcomes between standard instructor-led classroom-based CPR training with the alternative training methods among laypersons.

## Methods

### Study design

A research question was identified using the PICO strategy (Population (P): laypersons not trained in CPR, Intervention (I): alternative CPR training methodologies, Comparison (C): standard CPR training methodology, Outcome (O): CPR knowledge, quality, and skill performance). After establishing the research domain, inclusion and exclusion criteria were established to identify and select relevant articles. After assessing the quality of the studies included, data was extracted, organized, summarized, and charted accordingly. The results were analyzed and reported. The primary research question guiding this review is: “What are the differences in CPR knowledge, skill performance, and quality in laypersons receiving alternative CPR training when compared to standard training methodology?”

### Search strategies

We searched PubMed or Medical Literature Analysis and Retrieval System Online (Medline), and Google Scholar for relevant articles from January 1995 to May 2020. Medical subject headings (MeSH) were searched using Boolean operators “*OR/AND*”. The search terms were: (“hands-only CPR” OR “cardiopulmonary resuscitation” OR “CPR”) AND (“teaching methodologies” OR “training methods”) AND (“medical students” OR “bystanders” OR “laypersons” OR “health-care workers” OR “school children” OR “physicians” OR “nurses” OR “paramedical staff” OR “technicians”).

### Inclusion and exclusion criteria

We included studies which compared two or more CPR training methodologies targeting laypersons with no previous CPR training. Studies describing a single methodology with no comparison group were excluded as were the case reports, case series, and non-English articles.

### Identification and selection of studies

**Fig. 1:**
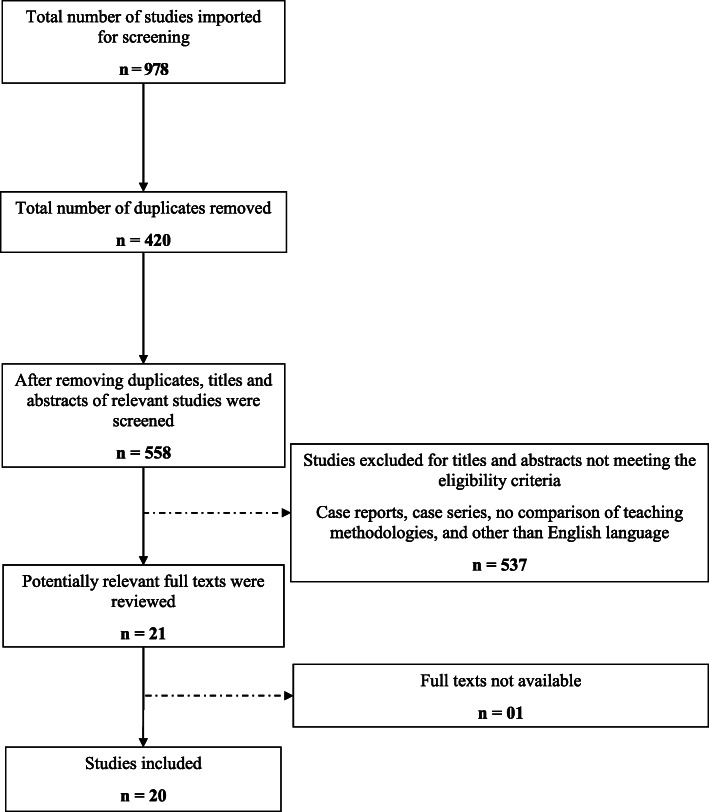
PRISMA Flow diagram for database search of studies

The studies were selected after two stages of screening. Two researchers (DMA and BH) independently, extracted data. In stage 1, we screened the article titles and abstracts and those which matched the inclusion criteria were selected for full text review. In the final stage, we reviewed full texts of the articles and determined their inclusion in this review. Any conflicts between researchers during the article screening process was resolved by the senior researcher (JR). Data was organized using a simple database on Microsoft Excel. Figure 1 presents a Preferred Reporting Items for Systematic Reviews and Meta-Analyses (PRISMA) flow diagram showing the process of searching and selecting the research articles.

### Data extraction from included studies

After article selection, we extracted and recorded data in a data extraction form in an excel spreadsheet. The domains in the data extraction form were: year and country of publication, intervention tested, study design, sample size, study population, presence of prior training, outcome measures, and key findings.

### Quality assessment of studies

The quality was assessed using the Effective Public Health Practice Project (EPHPP) Quality Assessment Tool [18]. Two researchers (DMA and BH) reviewed each study using EPHPP. The results of the quality assessment are summarized in Table 2. No studies were excluded on the basis of quality assessment, as this quantitative evidence synthesis aimed to include all articles relevant to our review question.

### Summarizing the findings

We summarized our findings into the following research domains: standard instructor-led classroom-based CPR training, non-standard face to face CPR training, hybrid CPR training, and online CPR training.

### Definition of terms

The definitions of commonly used terminologies in this article are detailed in Table 1.

**Table 1:** Definitions of training methodologies employed to train participants

Terminology	Definition
Alternative CPR Training	CPR teaching methodologies including non-standard face to face, hybrid, and online CPR training
Flipped CPR learning	CPR training in which participants watch short pre-recorded videos followed by hands-on practice with an instructor
Flowchart-supplemented CPR Training	Provision of a flowchart prior to beginning resuscitation attempts
Hybrid CPR Training	CPR training using a combination of face to face and online teaching methodologies. The examples include Kiosk session, interactive computer training plus instructor-led training, and video learning followed by hands-on CPR training.
Jigsaw Model CPR Training	Division of the intervention group randomly into a chest compression and a ventilation group
Kiosk Session	Features a touch screen with a video program that gives a brief “how-to” followed by a practice session and a CPR test
Multistaged Approach	A three-staged approach comprising of a bronze (50 compressions only), silver (50 compressions: 5 breaths), and a gold (conventional CPR) stage
Non-standard Face to Face CPR Training	Face to face CPR training conducted without using an expert instructor-led teaching methodology or standard CPR training material. The examples include simplified (hands only) CPR training, peer-based CPR training, jigsaw model CPR training, flowchart-supplemented CPR training, and multistage CPR training.
Online CPR Training	Digital CPR training using video self-instruction, interactive computerized module, voice advisory mannequin feedback, or virtual reality
Peer-based CPR Training	Training received by a group of participants who have been instructed by professional instructors in advance
Simplified (hands only) CPR Training	Simplifying the learning of CPR by focusing on continuous chest compressions with simple hand placement
Standard CPR Training	An instructor-led CPR training conducted in a classroom setting
Virtual Reality CPR Training	CPR training in a simulated environment using smartphones, headphones, and virtual reality goggles with the mobile App providing feedback
Voice Advisory Mannequin Feedback Training	An immediate, standardized, and corrective audio feedback training without presence of an instructor

## Results

### Studies’ characteristics

A total of 978 articles were retrieved from PubMed and Google Scholar. Four hundred and twenty duplicate articles were excluded. Out of the remaining 558 articles, 537 articles were either not comparing different teaching methodologies, were case reports or case series, or were written in a language other than English, and no English translation was available and therefore were excluded. Among the remaining 21 articles, 1 articles did not have available full texts. Twenty full-text articles were reviewed and included in this study. Out of these twenty articles, ten had a moderate global rating, while ten had a strong global rating based on Effective Public Health Practice Project (EPHPP) Quality Assessment Tool (Table 2**)**.

**Table 2:** Results of quality assessment of included studies using the Effective Public Health Practice Project (EPHPP) tool

First Author, Country, and Year	Selection Bias	Study Design	Confounders	Blinding	Data Collection Methods	Withdrawal and Dropouts	Global Rating
**Standard versus Non-standard Face to Face CPR Training**							
Ko RJM Singapore, 2018	Strong	Strong	Strong	Moderate	Strong	Moderate	**Strong**
Beck S Germany, 2015	Strong	Strong	Strong	Weak	Moderate	Moderate	**Moderate**
Charlier N Belgium, 2016	Moderate	Strong	Strong	Weak	Strong	Moderate	**Moderate**
Rossler B Austria, 2013	Strong	Strong	Strong	Weak	Moderate	Moderate	**Moderate**
Chamberlain D UK, 2001	Strong	Strong	Strong	Weak	Moderate	Moderate	**Moderate**
Choi HS Korea, 2015	Strong	Moderate	Strong	Moderate	Weak	Moderate	**Moderate**
**Standard versus Hybrid CPR Training**							
Nakanishi T Japan, 2017	Moderate	Moderate	Strong	Strong	Moderate	Strong	**Strong**
Heard DG USA, 2019	Strong	Strong	Strong	Weak	Moderate	Moderate	**Moderate**
Reder S USA, 2006	Strong	Strong	Strong	Weak	Moderate	Moderate	**Moderate**
**Standard versus Online CPR Training**							
Todd KH USA, 1998	Moderate	Strong	Strong	Moderate	Moderate	Moderate	**Strong**
Rehberg RS USA, 2009	Moderate	Strong	Strong	Weak	Strong	Moderate	**Moderate**
Beskind DL USA, 2016	Strong	Strong	Strong	Weak	Strong	Moderate	**Moderate**
Todd KH USA, 1999	Moderate	Strong	Strong	Moderate	Strong	Moderate	**Strong**
Ahn JY Korea, 2011	Strong	Moderate	Strong	Moderate	Moderate	Moderate	**Strong**
Kardong-Edgren SE USA, 2010	Moderate	Moderate	Strong	Weak	Strong	Moderate	**Moderate**
Diez N Spain, 2013	Moderate	Strong	Strong	Strong	Strong	Moderate	**Strong**
Ali S India, 2019	Moderate	Strong	Strong	Moderate	Strong	Strong	**Strong**
Isbye DL Denmark, 2006	Strong	Moderate	Strong	Strong	Strong	Strong	**Strong**
Nas J Netherland, 2020	Strong	Strong	Strong	Strong	Strong	Strong	**Strong**
Einspruch EL USA, 2007	Strong	Strong	Strong	Strong	Strong	Strong	**Strong**

### Research domains

Among the twenty studies included in this review, eleven compared online CPR training with the standard training, six studies compared non-standard face to face CPR training with the standard training, and three studies compared the standard CPR training with hybrid training methodologies. Among the included studies, fourteen studies were randomized controlled trials, two had an interventional study design, two were cluster randomized controlled trials, and two studies had a case-control study design. The study population comprised of school children, laypersons, medical students, and nursing students. The details of individual studies are summarized in Table 3.

**Table 3:** Summarized findings of included CPR training methodology research articles

Year and Country	Intervention Tested	Study Design	Sample Size	Target Group	Prior Training	Outcome Measures	Key Findings
Standard versus Non-standard Face to Face CPR Training							
Singapore 2018 [19]	Simplified vs. standard CPR	Randomized Controlled Trial	85	Layperson	No	CPR quality	Simplified CPR group followed algorithm better (*p <* 0.01), had higher number and proportion of adequate compressions (*p <* 0.01), and had shorter hands-off time (*p <* 0.001).
Germany 2015 [20]	Peer-instructor vs. professional instructor	Randomized Controlled Trial	1087	School Children	No	CPR performance	Similar CPR performance between groups (40.3% vs. 41.0%).
Belgium 2016 [21]	Peer-based (jigsaw model) vs. expert instructor	Randomized Controlled Trial	137	School Children	No	CPR performance	All groups met European Resuscitation Council 2010 guideline. Chest compression depth different between ventilation vs. compression group (*p <* 0.01).
Austria 2013 [22]	Flowchart supported training	Randomized Controlled Trial	83	Layperson	No	CPR performance and quality	Flowchart group showed shorter hands-off time (147 s vs. 169 s, *p =* 0.024) and more confidence (7 vs. 5, *p =* 0.0009) but had longer time to chest compression (60s vs. 23 s, *p <* 0.0001).
UK 2001 [23]	Three-stage vs. conventional training	Randomized Controlled Trial	495	Layperson	No	CPR quality and knowledge	In first 8 min, using 50:5 ratio, 58% more compressions can be made. Staged group had better ‘shout for help’ after 2 months (*p =* 0.02 to *p <* 0.01) and adequate compressions after retraining (*p =* 0.05) and at 4 months (*p =* 0.04).
Korea 2015 [24]	Peer-assisted learning vs. professional instructor training	Prospective Case-Control Study	187	High-school Students	No	CPR performance and knowledge	No difference in willingness to perform CPR (64.7% vs. 55.2%, *p =* 0.202) and knowledge retention (61.76 ± 17.80 vs. 60.78 ± 39.77, *p =* 0.848) between peer-assisted and professional instructor groups.
Standard versus Hybrid CPR Training							
Japan 2017 [25]	Coventional vs. flipped learning	Interventional Study	108	Medical Students	No	CPR quality	No difference in time to first chest compression (33 s vs. 31 s, *p =* 0.73) or number of chest compressions (101.5 vs. 104, *p =* 0.75).
USA 2019 [26]	Traditional vs. video-only vs. video + hands-on session at a Kiosk	Randomized Controlled Trial	738	layperson	No	CPR performance and quality	After the initial education session, the video-only group had a lower total score (compressions correct on hand placement, rate, and depth) (−9.7; 95% confidence interval [CI] -16.5 to −3.0) than the classroom group. There were no significant differences on total score between classroom and kiosk participants.
USA 2006 [27]	Interactive-computer training and interactive-computer training plus instructor-led (hands-on) practice vs. traditional training	Cluster Controlled Trial	784	High School Students	No	CPR performance and knowledge	For all outcome measures mean scores were higher in the instructional groups than in the control group. Two days after training all instructional groups had mean CPR and AED knowledge scores above 75%, with use of the computer program scores were above 80%.
Standard versus Online CPR Training							
USA 1998 [28]	Heartsaver CPR training (traditional) vs. video self instruction	Prospective Randomized Controlled Trial	89	Incoming Freshmen Medical Graduates	No	CPR performance	VSI trainees displayed superior overall performance compared with traditional trainees. Twenty of 47 traditional trainees (43%) were judged not competent in their performance of CPR, compared with only 8 of 42 VSI trainees (19%; absolute difference, 24%; 95% confidence interval, 5 to 42%).
USA 2009 [29]	Traditional (group 1) vs. online (group 2 - computerized module with video) version	Randomized Controlled Trial	64	Undergrad Freshmen	No	CPR quality and knowledge	On the standardized knowledge examination and skill performance evaluation, Group 2 scored lower than Group 1; however, no statistically significant difference between the groups existed. MANOVA indicated there was a significant difference in the quality of CPR compressions (location, rate, depth, and release), ventilation rate and volume.
USA 2016 [30]	Brief video vs. traditional training	Cluster Randomized Trial	179	School Children	No	CPR quality	At post-intervention and 2 months, BV and CCO class students called 911 more frequently and sooner, started chest compressions earlier, and had improved chest compression rates and hands-off time compared to baseline.
USA 1999 [31]	Video self instruction vs. traditional CPR training	Randomized Controlled Trial	190	Layperson	No	CPR performance and knowledge	VSI trainees displayed a comparable level of performance to that achieved by traditional trainees. Observers scored 40% of VSI trainees competent or better in performing CPR, compared with only 16% of traditional trainees (absolute difference 24, 95% confidence interval 8 to 40%).
Korea 2011 [32]	Video based vs. traditional training	Single-Blind Case-Control Study	75	Students	No	CPR performance	Three months after initial training, the video-reminded group showed more accurate airway opening (*P <* 0.001), breathing check (*P* < 0.001), first rescue breathing (*P =* 0.004), and hand positioning (*P =* 0.004) than controls. They also showed significantly higher self-assessed CPR confidence scores and increased willingness to perform bystander CPR in cardiac arrest than the controls at 3 months (*P <* 0.001 and *P* = 0.024, respectively).
USA 2010 [33]	HeartCode™BLS with VAM vs. instructor-led training	Randomized Controlled Trial	604	Nursing Students	No	CPR quality	No difference in compression rate between groups. HeartCode™BLS with VAM group had more compressions with adequate depth and correct hand placement, and had more ventilations with adequate volume.
Spain 2013 [34]	Voice Advisory Mannequin vs. instructor training	Randomized Controlled Trial	43	Medical Students	No	CPR performance	VAM group performed more correct hand position (73% vs. 37%, *p* = 0.014) and had better compression rate (124/min vs. 135/min, *p =* 0.089). Women in VAM group showed improvement in compression depth (36 mm to 46 mm, *p* = 0.018) and percentage of insufficient compressions (56 to 15%, *p* = 0.021) after training.
India 2019 [35]	Video-based CPR training vs. instructor-based CPR training	Randomized Controlled Trial	109	Undergrad University Students	No	CPR performance	Video-based group performed better scene safety (95.2% vs. 76.1%) and call for help (97.6% vs. 76.1%) than the instructor-based group (*p* < 0.05). Moreover, the video-based group had shorter response to compression time (35 ± 9 s vs. 54 ± 14 s) as compared to the instructor-based group (*p* < 0.001).
Denmark 2006 [36]	DVD-based self training vs. instructor training	Interventional Study	238	Layperson	No	CPR knowledge	After 3 months, no significant difference in total scores of CPR performance between groups. The instructor group had better score in assessment of breathing (91% vs. 72%) as compared to the DVD-based group (*p* = 0.03). However, DVD-based group had better average inflation volume (844 ml vs. 524 ml, *p =* 0.006) and chest compression depth (45 mm vs. 39 mm, *p =* 0.005).
Netherland 2020 [37]	Virtual reality CPR training vs. face-to-face CPR training	Randomized Controlled Trial	381	Layperson	No	CPR performance	The VR group was inferior to face-to-face training in chest compression depth (49 mm vs. 57 mm), chest compression fraction (61% vs. 67%, *p* < 0.001), proportion of participants fulfilling depth (51% vs. 75%, *p <* 0.001), and rate requirements (50% vs. 63%, *p =* 0.01), but superior in chest compression rate (114/min vs. 109/min) and compressions with full release (98% vs. 88%, *p =* 0.002). The VR group had lower overall scores (10 vs. 12, *p <* 0.001) as compared to the face-to-face group.
USA 2007 [38]	Video self-training vs. instructor training	Randomized Controlled Trial	285	Layperson	No	CPR performance and knowledge	Immediately post-training, video group had higher scores in overall performance (60% vs. 42%), assessing responsiveness (90% vs. 72%), ventilation volume (61% vs. 40%), and correct hand placement (80% vs. 68%) but lower scores in calling 911 (71% vs. 82%). At 2 months post-training, video group had higher scores in overall performance (44% vs. 30%), assessing responsiveness (77% vs. 60%), ventilation volume (41% vs. 36%), and correct hand placement (64% vs. 59%) but lower scores in calling 911 (53% vs. 74%).

### Characteristics of different CPR training methodologies

**Fig. 2:**
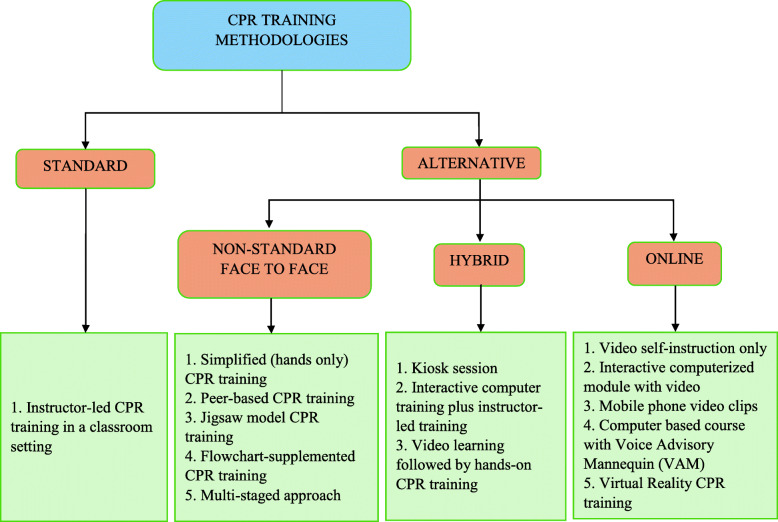
Comparison of the mode of delivery of different CPR training methodologies

The CPR training methodologies were divided into two broad categories including standard instructor-led classroom-based CPR training and alternative CPR training. The alternative CPR teaching methodology was further classified as non-standard face to face CPR training, hybrid CPR training, and online CPR training. The comparison of content, duration, mode of delivery, standard of content, and measured outcomes between different training methodologies are detailed in Fig. 2 and Table 4. Significant difference was noted between the duration of the teaching methods. The studies reported a longer duration of standard CPR training (20 min to 6 h) when compared to non-standard face to face (45 min to 3 h), hybrid (4 min to 1.5 h), and online CPR training methods (1 min to 1.5 h). Moreover, variability was also noted in the standard of content taught between different training methods and within each training method as well. Although “Einlebenretten” (“save one life”) educational framework [20] and European Resuscitation Council (ERC) 2010 guidelines [21, 34] were the two contents similar between standard and non-standard face to face CPR training, the standard training group also used contents from ERC 2005 guidelines [34], American Heart Association (AHA) Heartsaver Citizen CPR course [27, 28, 31, 38], AHA 2010 guidelines [25], National Safety Council Adult CPR training program [29], HeartCode BLS course [33], Dutch Resuscitation Council course [37], and Danish Red Cross course [36]. Although the computer-based HeartCode BLS course [33] and National Center for Early Defibrillation course [27] were similar between hybrid and online CPR training methodology, the standard of content was also adopted from other sources in these instructional methods. The hybrid teaching methodology had contents from Japanese Red Cross Society [25] and AHA 2010 guidelines [25], while online training method adopted content from National Safety Council Adult CPR training program [29] and TrygFonden foundation [36]. The content (CPR, ventilation, and breathing) and outcomes measured (CPR performance, quality, knowledge, attitude, self-confidence, and willingness to perform CPR) were similar between the training methodologies.

**Table 4:** A comparison between the characteristics of different CPR training methodologies

Variables	Standard CPR Training	Alternative CPR Training		
		Non-standard Face to Face CPR Training	Hybrid CPR Training	Online CPR Training
Content^a^	CPR, ventilation, and breathing	CPR, ventilation, and breathing	CPR, ventilation, and breathing	CPR, ventilation, and breathing
Duration	20 min - 6 h	45 min - 3 h	4 min – 1.5 h	1 min – 1.5 h
Mode of delivery	Professional instructor-led classroom based CPR training	Peer-based, flowchart-supplemented, simplified, and multi-staged CPR training	Kiosk session, interactive-computer based training plus instructor-led practice, and video-learning followed by hands-on CPR training	Interactive-computer based, video-self instruction only, mobile phone video clips, computer based course with Voice Advisory Mannequin (VAM), and virtual reality CPR training
Content Standard	1. “Einlebenretten” (“save one life”) educational framework 2. European Resuscitation Council (ERC) 2005 and 2010 guidelines 3. American Heart Association (AHA) Heartsaver Citizen CPR course 4. American Heart Association (AHA) 2010 guidelines 5. National Safety Council Adult CPR training program 6. HeartCode BLS course 7. Dutch Resuscitation Council 8. Danish Red Cross	1. “Einlebenretten” (“save one life”) educational framework 2. European Resuscitation Council (ERC) 2010 guidelines	1. Computer-based HeartCode BLS course 2. National Center for Early Defibrillation 3. Japanese Red Cross Society 4. American Heart Association (AHA) 2010 guidelines	1. Computer-based HeartCode BLS course 2. National Safety Council Adult CPR training program 3. National Center for Early Defibrillation 4. TrygFonden foundation (Denmark)
Skill taught^a^	Calling for help, checking breathing, appropriate number and adequate depth of chest compressions, correct hand placement, compression to ventilation ratio, and adequate ventilation	Calling for help, checking breathing, appropriate number and adequate depth of chest compressions, correct hand placement, compression to ventilation ratio, and adequate ventilation	Calling for help, checking breathing, appropriate number and adequate depth of chest compressions, correct hand placement, compression to ventilation ratio, and adequate ventilation	Calling for help, checking breathing, appropriate number and adequate depth of chest compressions, correct hand placement, compression to ventilation ratio, and adequate ventilation
Outcomes measured^a^	1. CPR skill performance = compression depth, hand position, adequacy of chest recoil, volume of ventilation 2. CPR quality = time to initiate CPR, continuous chest compressions, number and adequacy of compressions, hand placement, hands-off time 3. CPR knowledge = acquisition and retention 4. CPR related attitudes 5. Self-confidence and willingness to perform CPR	1. CPR skill performance = compression depth, hand position, adequacy of chest recoil, volume of ventilation 2. CPR quality = time to initiate CPR, continuous chest compressions, number and adequacy of compressions, hand placement, hands-off time 3. CPR knowledge = acquisition and retention 4. CPR related attitudes 5. Self-confidence and willingness to perform CPR	1. CPR skill performance = compression depth, hand position, adequacy of chest recoil, volume of ventilation 2. CPR quality = time to initiate CPR, continuous chest compressions, number and adequacy of compressions, hand placement, hands-off time 3. CPR knowledge = acquisition and retention 4. CPR related attitudes 5. Self-confidence and willingness to perform CPR	1. CPR skill performance = compression depth, hand position, adequacy of chest recoil, volume of ventilation 2. CPR quality = time to initiate CPR, continuous chest compressions, number and adequacy of compressions, hand placement, hands-off time 3. CPR knowledge = acquisition and retention 4. CPR related attitudes 5. Self-confidence and willingness to perform CPR

^a^The content, skills taught, and outcomes measured were similar between standard and alternative CPR trainings

### Comparison of outcomes between different training methodologies

The studies assessed three main outcomes after CPR training which included CPR skill performance, CPR quality, and CPR knowledge. The difference in each outcome was compared between the standard instructor-led classroom-based CPR training and alternative (non-standard face to face, hybrid, and online) CPR training methodologies. The detailed description of these differences is illustrated in Table 5.

**Table 5:** Comparison between standard CPR training versus non-standard face to face, hybrid, and online CPR teaching methodologies

	Alternative CPR Training		
	Non-standard Face to Face CPR Training	Hybrid CPR Training	Online CPR Training
Standard CPR Training (Instructor-led Classroom-based)	**CPR Performance** **1.** Similar performance was seen between the peer-led (41.0%, *N* = 466) and instructor-led (40.3%, *N* = 471) groups [20]. **2.** No significant difference between jigsaw and instructor-led group. Chest compression depth was different between ventilation and compression groups (*p* < 0.01) [21]. **3.** Flowchart group was more confident than non-flowchart group (7 ± 2 vs. 5 ± 2, *p =* 0.0009) [22]. **4.** No difference in willingness to perform CPR (64.7% vs. 55.2%, *p* = 0.202) between peer-assisted and professional instructor groups [24].	**CPR Performance** **1.** The kiosk group outperformed instructor-led group on hand placement (4.9) but not on compression depth score (− 5.6) [26]. **2.** For all outcome measures, mean scores were higher in the interactive-computer training plus instructor-led practice as compared to the instructor-led group [27].	**CPR Performance** **1.** Video self-instruction group had superior overall performance with only 19% non-competent trainees in comparison to 43% non-competent trainees in the instructor-led group [28]. **2.** Forty percent of video self-instruction trainees were competent compared to 16% competent in the instructor-led group [31]. **3.** Video group had more accurate airway opening (*p* < 0.001), breathing check (*p <* 0.001), first rescue breathing (*p* = 0.004), hand positioning (*p =* 0.004), and higher confidence and willingness to perform CPR at 3 months [32]. **4.** Voice advisory mannequin feedback group performed more correct hand position (73% vs. 37%, *p* = 0.014) and better compression rate (124 vs 135, *p* = 0.089) than the instructor-led group. Women in the voice advisory mannequin feedback group showed more improvement in compression depth (*p* = 0.018) and adequate compressions (*p* = 0.021) [34]. **5.** The video-only group had lower compression depth scores (− 9.9) than the classroom group [26]. **6.** For all outcome measures, mean scores were higher in the interactive-computer training as compared to the instructor-led group [27]. **7.** Video-based group performed better scene safety (95.2% vs. 76.1%) and call for help (97.6% vs. 76.1%) than the instructor-based group (*p <* 0.05). Moreover, the video-based group had shorter response to compression time (35 ± 9 s vs. 54 ± 14 s) as compared to the instructor-based group (*p <* 0.001) [35]. **8.** The VR group was inferior to face-to-face training in chest compression depth (49 mm vs. 57 mm), chest compression fraction (61% vs. 67%, *p <* 0.001), proportion of participants fulfilling depth (51% vs. 75%, *p <* 0.001), and rate requirements (50% vs. 63%, *p =* 0.01), but superior in chest compression rate (114/min vs. 109/min) and compressions with full release (98% vs. 88%, *p* = 0.002). The VR group had lower overall scores (10 vs. 12, *p <* 0.001) as compared to the face-to-face group [37]. **9.** Immediately post-training, video group had higher scores in overall performance (60% vs. 42%), assessing responsiveness (90% vs. 72%), ventilation volume (61% vs. 40%), and correct hand placement (80% vs. 68%) but lower scores in calling 911 (71% vs. 82%) as compared to instructor-led training [38].
Standard CPR Training (Instructor-led Classroom-based)	**CPR Quality** **1.** Simplified CPR group performed better on the algorithm (*p <* 0.01), had higher number and adequate compressions (*p <* 0.01), and shorter hands-off time (*p <* 0.001). No difference in time taken to initiate CPR [19]. **2.** Shorter hands-off time in the flowchart (147 ± 30s) versus non-flowchart group (169 ± 55 s) (*p* = 0.024). However, time to chest compression was longer in the flowchart group (60 ± 24 s vs. 23 ± 18 s, *p* < 0.0001) [22]. **3.** 58% more compressions can be achieved with a silver-staged approach (50:5 ratio) in the first 8 critical minutes. Staged group had better ‘shout for help’ after 2 months (*p =* 0.02 to *p <* 0.01) and adequate compressions after retraining (*p* = 0.05) and at 4 months (*p* = 0.04) [23].	**CPR Quality** **1.** No statistically significant difference in time to first chest compression (33 s vs. 31 s, U = 1171, *p* = 0.73) and number of total chest compressions (101.5 vs. 104, U = 1083, *p* = 0.75) between the instructor-led and flipped learning group, respectively [25]. **2.** There was no significant difference on total scores between instructor-led and kiosk participants [26].	**CPR Quality** **1.** The instructor-led training group showed superior performance than the computer-based training group in the quality of CPR compressions (location, rate, depth, and release) [29]. **2.** Both brief video and instructor-led group called 911 more frequently and sooner, started chest compression earlier, and had improved chest compression rates and hands-off time. However, chest compression depth was better in the instructor-led versus the brief video group [30]. **3.** Voice advisory mannequin feeback group had more compressions with adequate depth and hand placement, and had more ventilations with adequate volume than the instructor-led group. However, compression rates between the groups were similar [33]. **4.** The video-only group had a lower total score (compression rate, depth, and correct hand placement) (− 9.7) than the instructor-led group [26].
Standard CPR Training (Instructor-led Classroom-based)	**CPR Knowledge** **1.** Better retention was seen in the bronze (50 compressions) and silver (50 compressions:5 breaths) stages when compared to conventional training [23]. **2.** No difference in knowledge retention (61.76 ± 17.80 vs. 60.78 ± 39.77, *p* = 0.848) between peer-assisted and professional instructor groups [24].	**CPR Knowledge** **1.** Mean CPR knowledge was above 80% with use of a computer program two days after training [27].	**CPR Knowledge** **1.** Although the computer-based training group had lower scores, there was no significant difference from the instructor-led training group [29]. **2.** Video self-instruction trainees and instructor-led trainees achieved comparable scores on CPR-related knowledge and attitudes [31]. **3.** Mean CPR knowledge was above 80% with use of a computer program two days after training [27]. **4.** After 3 months, the instructor group had better score in assessment of breathing (91% vs. 72%) as compared to the DVD-based group (*p* = 0.03). However, DVD-based group had better average inflation volume (844 ml vs. 524 ml, *p* = 0.006) and chest compression depth (45 mm vs. 39 mm, *p* = 0.005) [36]. **5.** At 2 months post-training, video group had higher scores in overall performance (44% vs. 30%), assessing responsiveness (77% vs. 60%), ventilation volume (41% vs. 36%), and correct hand placement (64% vs. 59%) but lower scores in calling 911 (53% vs. 74%) [38].

#### Standard versus non-standard face to face CPR training

The non-standard face to face CPR training included simplified (hands-only) CPR, peer-based CPR training, Jigsaw model CPR training, flowchart-supplemented CPR training, and a multi-staged approach to CPR training. Out of the twenty studies, five randomized controlled trials and one prospective case-control study fell under this domain. Two studies compared CPR performance and one study compared CPR quality. More than one outcome was measured by three studies in which one study compared CPR performance and quality, one study compared CPR quality and knowledge, and one study compared CPR performance and knowledge between the instructional methods.

In CPR performance, no statistically significant difference was noted between the peer-led (41.0%, *N* = 466), jigsaw model group, and the standard instructor-led group (40.3%, *N* = 471) [20, 21]. Moreover, willingness to perform CPR was also similar between the peer-led (64.7%) and standard instructor-led group (55.2%, *p* = 0.202) [24]. However, flowchart supplemented group (7 ± 2) was more confident in performing CPR than the instructor-led group (7 ± 2 vs. 5 ± 2, *p* = 0.0009) [22].

In CPR quality, the simplified CPR group performed better on CPR algorithm (*p* < 0.01), had higher number and adequate chest compressions (*p <* 0.01), and shorter hands-off time (*p* < 0.001) when compared with the standard training group [19]. Although the flowchart-supplemented group showed shorter hands-off time (147 ± 30s vs. 169 ± 55 s, *p* = 0.024), the time to chest compression was longer (60 ± 24 s vs. 23 ± 18 s, *p* < 0.0001) as compared to the instructor-led group [22]. The staged CPR group had better “shout for help” (*p =* 0.02 to *p <* 0.01) and more adequate compressions (*p* = 0.05 to *p* = 0.04) when compared to standard training [23].

Although better CPR knowledge retention was seen in the multi-staged approach CPR training when compared to the standard group [23], no difference in retention was seen between peer-assisted (61.76 ± 17.80) and professional instructor groups (60.78 ± 39.77, *p* = 0.848) [24].

#### Standard versus hybrid CPR training

The hybrid CPR training included a kiosk group, an interactive computer training group plus an instructor-led training group, and a video learning group followed by hands-on CPR training. Three studies fell under this domain. One study compared CPR quality, while one study compared CPR performance and quality and one study compared CPR performance and knowledge between the instructional methods.

In CPR performance, although the kiosk group outperformed the instructor-led group on hand placement (+ 4.9), they scored lower on compression depth (− 5.6) [26]. Moreover, for all outcome measures, mean scores were higher in the interactive-computer training group plus instructor-led practice group when compared to the instructor-led group [27].

In terms of CPR quality, no significant difference was noted in time to first chest compression (33 s vs. 31 s, U = 1171, *p* = 0.73) and number of total chest compressions (101.5 vs. 104, U = 1083, *p* = 0.75) between the instructor-led group and flipped learning group [25]. Furthermore, the kiosk group and the instructor-led group had similar total scores after training [26].

Lastly, use of a computer program resulted in higher knowledge retention (80%) as compared to the instructor-led group (75%) two days after training [27].

#### Standard versus online CPR training

The online CPR training methodology included video self-instruction, interactive computerized module with video, mobile phone video clips, a computer-based course with Voice Advisory Mannequin (VAM), and virtual reality CPR training. Eleven studies fell under this domain. Five studies compared CPR performance, two studies compared CPR quality, and one study compared CPR knowledge between the instructional methods. More than one outcome was compared by three studies in which, two studies compared CPR performance and knowledge while one study compared CPR quality and knowledge between instructional methods.

In CPR performance, video self-instruction group had superior overall performance scores with only 19% non-competent trainees as compared to 43% non-competent trainees in the instructor-led group [28]. Moreover, another study also reported similar findings in which, 40% of the video self-instruction group were competent when compared to only 16% competency in the instructor-led group [31]. The group which received video-based training also had more accurate airway opening (*p* < 0.001), breathing check (*p <* 0.001), first rescue breathing (*p* = 0.004), hand positioning (*p =* 0.004), and higher confidence and willingness to perform CPR at 3 months when compared to the instructor-led group [32]. Furthermore, another study showed that the video-based group performed better scene safety (95.2% vs. 76.1%, *p <* 0.05), call for help (97.6% vs. 76.1%, *p <* 0.05), and had shorter response to compression time (35 ± 9 s vs. 54 ± 14 s, *p <* 0.001) as compared to the standard instructor-based group [35]. A study in United States showed higher overall performance (60% vs. 42%), appropriate responsiveness assessment (90% vs. 72%), adequate ventilation volume (61% vs. 40%), and correct hand placement (80% vs. 68%) in the video group as compared to instructor-led training [38]. However, one study reported lower compression depth scores (− 9.9) [26] while another study had lower scores in calling 911 (71% vs. 82%) [38] in the video group as compared to the instructor-led group. Voice Advisory Mannequin (VAM) feedback was another methodology adopted for online training in one of the studies and those participants trained using this method had more correct hand position (73% vs. 37%, *p* = 0.014) and better compression rate (124 vs 135, *p* = 0.089) than the instructor-led group [34]. A study in Netherlands compared standard instructor-led training with Virtual Reality (VR) CPR teaching methodology. Although the VR group had better chest compression rates (114/min vs. 109/min) and proportion of compressions with full release (98% vs. 88%, *p* = 0.002), the instructor-led group had higher overall scores (12 vs. 10, *p <* 0.001), better chest compression depth (57 mm vs. 49 mm), adequate chest compression fraction (67% vs. 61%, *p <* 0.001), higher proportion of participants fulfilling depth (75% vs. 51%, *p <* 0.001), and rate requirements (63% vs. 50%, *p =* 0.01) [37].

In CPR quality, the instructor-led training group had better quality of CPR compressions (location, rate, depth, and release) as compared to the computer-based training group [29]. Moreover, the chest compression depth was also better in the instructor-led group when compared to the group trained using brief videos [30]. Although the VAM feedback group showed similar compression rates, they had more compressions with adequate depth and hand placement, and had more ventilations with adequate volume than the instructor-led group [33].

Although some studies showed no significant difference in the CPR-related knowledge scores between the instructional methods [29, 31], other studies highlighted significant differences. A study in Denmark highlighted that after 3 months, although the DVD-based group had better average inflation volume (844 ml vs. 524 ml, *p* = 0.006) and chest compression depth (45 mm vs. 39 mm, *p* = 0.005), the instructor-led group was superior in assessment of breathing (91% vs. 72%) [36]. At 2 months post-training, another study illustrated that although the video group had higher scores in overall performance (44% vs. 30%), assessing responsiveness (77% vs. 60%), ventilation volume (41% vs. 36%), and correct hand placement (64% vs. 59%), the instructor-led group scored higher in calling 911 (74% vs. 53%) [38].

## Discussion

This is a comprehensive systematic review that compares CPR performance, quality, and knowledge between different teaching methodologies including standard instructor-led, non-standard face to face, hybrid, and online CPR trainings. This review includes 20 studies and 5961 participants and illustrates significant differences in both the characteristics and the outcomes between the instructional methodologies.

All the included articles had an experimental study design and had a moderate or strong global rating based on our quality assessment tool. Our results suggested that the standard instructor-led CPR training had a longer duration (20 min to 6 h) as compared to alternative CPR trainings (1 min to 3 h). Moreover, the standard of content also varied significantly between the instructional methods. Interestingly, our review also showed variability in the content within the standard instructor-led CPR training methodology in which the teaching material was adopted from multiple sources including “Einlebenretten” (“save one life”) educational framework [20], European Resuscitation Council (ERC) 2005 and 2010 guidelines [21, 34], American Heart Association (AHA) Heartsaver Citizen CPR course [27, 28, 31, 38], AHA 2010 guidelines [25], National Safety Council Adult CPR training program [29], HeartCode BLS course [33], Dutch Resuscitation Council course [37], and Danish Red Cross course [36].

The instructional methods were compared on the basis of CPR performance, quality, and knowledge which were the three primary outcomes of the studies. In CPR performance, when compared to the standard instructor-led CPR training, the non-standard face to face CPR trained group were although more confident in performing CPR [22], similar performance was seen in the peer-led [20, 24] and the jigsaw model groups [21]. Although the hybrid CPR training methodology led to higher overall performance scores including better hand placement, the instructor-led methodology outperformed on the chest compression depth scores [26, 27]. When compared to standard CPR training, online instructional methodology not only resulted in a higher percentage of competent trainees [28, 31], but it also resulted in more performance of scene safety, assessing responsiveness, calling for help, accurate airway opening, breathing check, first rescue breathing, adequate ventilation volume, shorter response to compression time, hand positioning, better compression rates, and higher confidence and willingness to perform CPR [32, 34, 35, 38]. However, instructor-led trainings had higher compression depth scores and higher scores in calling 911 when compared to online CPR training [26, 38]. With regards to CPR quality, the non-standard face to face CPR training methodology outperformed in the CPR algorithm, had higher “shout for help” rates, had better rate and quality of compressions, and had shorter hands-off time when compared with the standard training [19, 22, 23]. However, instructor-led groups took less time to start chest compressions [22]. The hybrid training groups and the instructor-led groups showed no statistically significant difference in the total obtained scores regarding CPR quality [25, 26]. When compared to standard CPR training, online instructional methods showed better hand position, better chest compression rates, shorter hands-off time, and more frequency of calling for help [29, 30, 39]. However, correct hand placement and adequate depth of chest compression was better in the instructor-led group [26, 30]. Lastly, when compared to standard CPR training, alternative instructional methods either had similar [24, 29, 31] or better knowledge retention [23, 27, 36, 38].

The results of our study can be explained by certain determining factors. Due to access to better technology and readily available training material nowadays, numerous alternative training methodologies are being tested and compared with the standard training to assess their efficacy. This constant testing and repetition of training results in constant improvement in these alternate training methodologies resulting in better outcomes among participants. However, the quality of CPR, particularly the adequacy of chest compressions, is still better among instructor-led group as technology to effectively monitor chest compression depth remotely is not widely available currently.

Our systematic review has certain implications. First, since the studies included in this review had a moderate or strong global rating, comparisons made between standard and alternative CPR instructional methods can be used for future trainings. Secondly, standard CPR training is resource intensive driven by availability of instructors and therefore has limited scalability. This is especially true in low resource settings where creating an organizational structure and large cadre of instructors to deliver courses may take longer times and require more resources. Our study highlights the feasibility of utilizing instructional technologies and also recognizes the shortcomings of using technology-only solutions. Thirdly, “standard” CPR training had significant variability in both the duration and the standard of content among different studies. It is important to create standards so that future methodologies can be measured and further innovative solutions can be developed. Given the risk of infection spread due to pandemics such as COVID-19, we believe that alternative to face-to-face teaching methodologies have significant promise and can be implemented safely and effectively to increase the rate and effectiveness of bystander CPR and in turn save more lives by strengthening the first component of the chain of survival. Future alternatives to face-to-face instruction including possibly remote monitoring of students may improve correct hand placement and adequate depth of chest compression.

### Limitations of the study

This article has some limitations. Most of the studies included in this review were conducted in developed countries and therefore, effective adaptability of alternate training methods in the local setting cannot be ascertained. Moreover, no study looked at CPR performance during an actual cardiac arrest event and none of the conducted studies measured the impact of different teaching methodologies on a population level. Furthermore, potential bias towards a particular CPR teaching methodology among trainers cannot be ruled out. Lastly, since no uniformity existed in the duration and content of standard CPR training, the outcomes cannot be compared with alternate training methods concretely enough.

## Conclusion

This review outlines that alternative CPR training methodologies are as effective or even possibly better when compared to standard in-person classroom CPR training in CPR performance and knowledge acquisition. However, effective CPR quality still largely depends on some in-person training. Due to promising results seen in alternate training methodologies and non-uniformity seen in standard instructional techniques, these instructional methods can be adopted as an alternative, particularly during this time of the COVID-19 pandemic. Moreover, future research should aim to develop uniformity in standard CPR training methodology, which will make comparison with alternative CPR instructional techniques more plausible.

## Data Availability

The data that support the findings of this study are available from the corresponding author upon reasonable request.

## Abbreviations

SCD: Sudden Cardiac Death
CPR: Cardiopulmonary Resuscitation
AHA: American Heart Association
COVID-19: Coronavirus disease
PRISMA: Preferred Reporting Items for Systematic Reviews and Meta-Analyses
EPHPP: Effective Public Health Practice Project
ERC: European Resuscitation Council
VAM: Voice Advisory Mannequin
VR: Virtual Reality
